# Electrical coupling between A17 cells enhances reciprocal inhibitory feedback to rod bipolar cells

**DOI:** 10.1038/s41598-018-21119-0

**Published:** 2018-02-15

**Authors:** Claudio Elgueta, Felix Leroy, Alex H. Vielma, Oliver Schmachtenberg, Adrian G. Palacios

**Affiliations:** 1 Centro Interdisciplinario de Neurociencia de Valparaíso, Universidad de Valparaíso, Valparaíso, Chile; 2 Physiology Institute I, Alberts Ludwig University, Freiburg, Germany; 3 Neuroscience department, Columbia University Medical Center, 1051 Riverside Drive, New York, NY 10032 USA

## Abstract

A17 amacrine cells are an important part of the scotopic pathway. Their synaptic varicosities receive glutamatergic inputs from rod bipolar cells (RBC) and release GABA onto the same RBC terminal, forming a reciprocal feedback that shapes RBC depolarization. Here, using patch-clamp recordings, we characterized electrical coupling between A17 cells of the rat retina and report the presence of strongly interconnected and non-coupled A17 cells. In coupled A17 cells, evoked currents preferentially flow out of the cell through GJs and cross-synchronization of presynaptic signals in a pair of A17 cells is correlated to their coupling degree. Moreover, we demonstrate that stimulation of one A17 cell can induce electrical and calcium transients in neighboring A17 cells, thus confirming a functional flow of information through electrical synapses in the A17 coupled network. Finally, blocking GJs caused a strong decrease in the amplitude of the inhibitory feedback onto RBCs. We therefore propose that electrical coupling between A17 cells enhances feedback onto RBCs by synchronizing and facilitating GABA release from inhibitory varicosities surrounding each RBC axon terminal. GJs between A17 cells are therefore critical in shaping the visual flow through the scotopic pathway.

## Introduction

The mammalian retina can elicit behavioral responses after the detection and processing of just a few photons^1^. This is accomplished by the scotopic pathway, a highly efficient microcircuit formed by distinct sets of dedicated excitatory and inhibitory cells^2–5^. Although the multiple synaptic steps that compose the scotopic pathway should theoretically add noise to the transmitted information, low-level luminous signals can faithfully be interpreted by the visual cortex^1^. One mechanism which is proposed to help in discriminating light-induced from randomly-generated spurious signals is the generation of a code of highly synchronized action potentials at the output of retinal ganglion cells (RGCs)^6–9^. Correlated firing can be supported by the existence of common convergent synaptic inputs with high release probability^6,10,11^ and by electrical coupling between RGCs or between populations of presynaptic neurons^6,12–14^. Therefore, synchronous activity could be a critical feature in earlier steps of retinal signal processing, allowing faithful transmission of weak luminous signals.

The second synapse of the main scotopic pathway is located between RBCs and AII amacrine cells. Glutamate released from RBC ribbon synapses depolarizes glycinergic AII cells, which in turn use the cone bipolar circuitry to convey scotopic signals towards RGCs^4,5,15,16^. RBCs also contact A17 amacrine cell dendrites forming a tripartite synapse with AII and A17 cells^17,18^. Glutamate from RBCs triggers GABA release from A17 cells onto RBC axon terminals^19^. This reciprocal feedback modulates the gain and kinetics of the RBC-AII synapse^20,21^ by curtailing RBC cell depolarization^22^. A17 cells receive inputs from around 100 RBCs, but each RBC makes only one contact with a given A17 cell^23–25^. The depolarization induced by a single RBC remains electrically confined at the postsynaptic varicosity thanks to the morphological and electrophysiological properties of A17 cells^23,26^. This arrangement could potentially introduce noise to the output of RBCs due to the variability between isolated reciprocal inhibitory synapses.

Interestingly, GABAergic A17 cells are known to be electrically interconnected^25,27–29^ and dendro-dendritic electrical coupling between neurons could support the homogenization of voltage fluctuations and enhance signal synchrony in downstream targets^30–33^. However, in spite of the central role of A17 cells in the scotopic circuitry^20,21^ and the relevance and prevalence of electrical synapses in this circuit^34–38^, a detailed characterization of how the intercellular coupling between A17 cells impacts signal processing in the scotopic pathway is still missing. In this study, using paired patch-clamp recordings and Ca^2+^ imaging we show how GJ-mediated coupling modulates the electrical properties and output of A17 cells to enhance reciprocal feedback onto RBCs and thereby control the flow of visual information through the scotopic pathway.

## Results

### GJ-mediated electrical coupling shapes A17 electrophysiological properties

**Figure 1:**
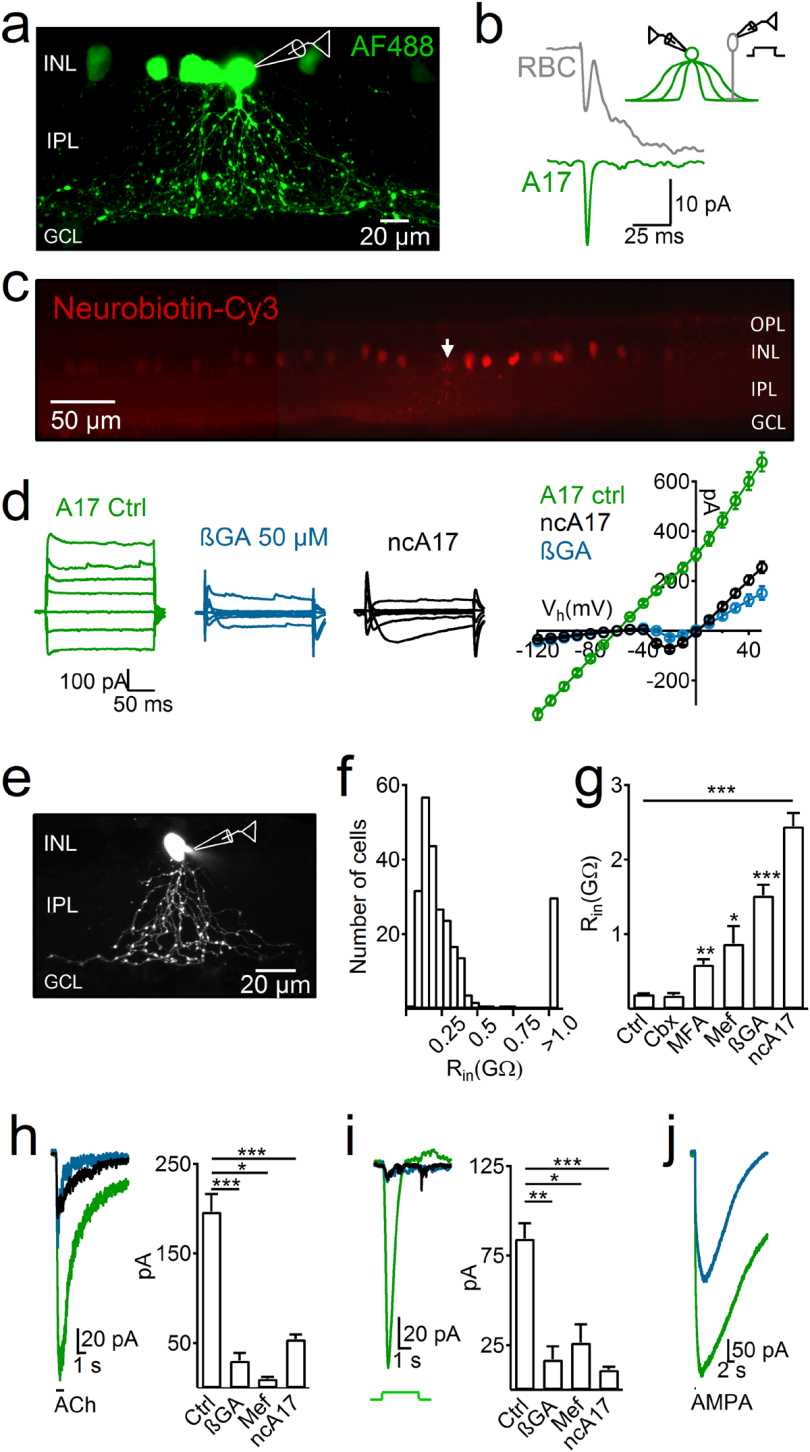
(**a**) Maximum intensity projection of confocal sections of an A17 AC after whole-cell patch-clamp recordings using Alexa Fluor 488 inside the recording pipette. Note dye coupling to other somas in the inner nuclear layer (INL). (**b**) Paired recording between a RBC and an A17 cell shows that after a voltage step (60 mV) in RBCs A17 cells receive a synaptic input (green trace) and provide GABAergic reciprocal feedback to the RBC (positive deflection in gray trace). (**c**) Montage of adjacent confocal images showing the spread of neurobiotin to neighboring cells after filling one A17 cell (white arrow). (**d**) Left representative traces of currents evoked by voltage steps from −100 to 20 mV (holding voltage −60 mV) in A17 cells in control conditions (green), after blocking gap junctions with 18β-glycyrrhetinic acid (βGA, blue traces) and in non-coupled A17s (ncA17s, black). Right shows the summary plot of 31, 12 and 21 experiments respectively. (**e**) Representative epifluorescence image of a ncA17 cell. Note lack of dye-coupling to other somas in the INL. (**f**) Histogram of the distribution of input resistance (R_in_) values of morphologically confirmed A17 cells. (**g**) Average R_in_ of A17 cells under control conditions (Ctrl), after perfusion with carbenexolone (Cbx 500 µM), meclofenamic acid (MFA 200 µM), mefloquine (Mef 50 µM) or 18-beta-glycyrrhetinic (βGA 50 µM), and of ncA17 cells. (**h**–**i**) Representative traces and summary plots of responses evoked by (**h**) puffs of acetylcholine (ACh 1 mM, 1 s), (**i**) light stimuli (530 nm, 3 s) or (**j**) puff-application of AMPA (200 µM, 100 ms) in A17 cells during control conditions (green), after block of gap junctions (blue) and in ncA17 cells (black traces). IPL, inner plexiform layer; ONL, outer nuclear layer; GCL, ganglion cell layer.

**Figure 2:**
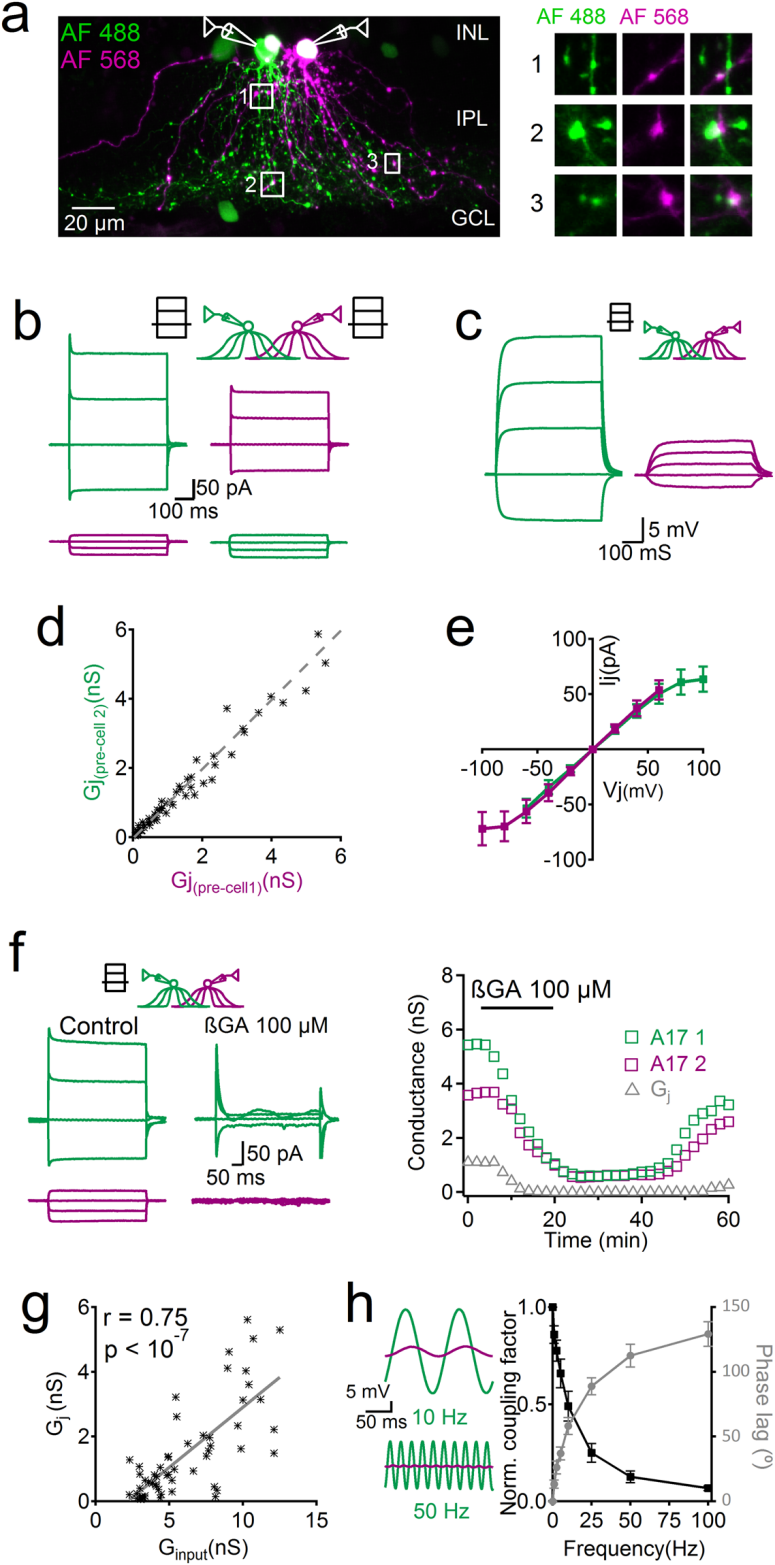
(**a**) Stack projection of two A17 cells after a paired patch-clamp recording. One pipette was filled with Alexa Fluor 488 (diffusing readily between cells) and the other with Alexa Fluor 568 (less permeable). Right panels show examples of putatively coupled varicosities. (**b**) Representative response of an A17 cell pair to voltage steps in each cell independently (from −80 mV to −40). Top traces show currents evoked in the stimulated cell and bottom panels the response in the paired cell. (**c**) Traces showing paired patch-clamp recording of A17 cells in current clamp mode. Current injection to the cell depicted in green induced voltage deflections of the same polarity in both cells. (**d**) Plot of calculated electrical junction conductivity (G_j_) depending on which A17 cell of individual pairs was stimulated. (**e**) Summary plot of gap junctional current of an A17 cell pair (Ij) as a function of the imposed junctional voltage (V_j_) (n = 34). (**f**) Representative traces (left) and time course of an experiment showing the effect of GJ block (βGA 100 µM) on junctional conductance between A17 cells (G_j_, gray trace) and their individual membrane conductance (G_input_, green and magenta traces,  see text for average data). (**g**) Plot showing the existence of a linear correlation between G_j_ and G_input_. (**h**) Left shows representative traces of the response to sinusoidal current injection in a pair of A17 cells. Summary graph on the right shows the relationship between frequency of injected waveform and the coupling factor normalized to the steady state coupling and the phase lag between signals in A17 cells. IPL, inner plexiform layer; INL, inner nuclear layer; GCL, ganglion cell layer.

Amacrine cells (AC) are a very heterogeneous population of cells^39–41^. To target A17 AC in acute retinal slices, we aimed at large oval somas at the border of the inner plexiform layer (IPL). After achieving whole-cell patch-clamp recordings their morphological features were revealed by the diffusion of fluorescent dyes from the recording pipette. A17 cells have thin dendrites that emerge from the soma, radially extending to the innermost border of the IPL and are densely embedded with varicose structures^29,42^ (Figs 1a, 2a). Correct identification of A17 cells was confirmed using paired recordings with RBCs. Upon depolarization of a RBC, we observed EPSCs in the A17 cell followed by reciprocal feedback in the RBC (n = 2, Fig. 1b). Out of the 252 patched cells that fulfilled the above mentioned morphological traits, the majority showed an almost linear I-V relationship (Fig. 1d), low input resistance (R_in_ = 0.2 ± 0.007 GΩ, n = 220), and were surrounded by other labeled somas within the inner nuclear layer (INL) when either Lucifer-Yellow, Alexa-Fluor 488 or neurobiotin were added to the intracellular solution (see methods; Figs 1a, 2a) confirming previous observations of dye coupling in rat A17 cells^29^. When neurobiotin was used, post-hoc analysis revealed a wide-ranging network of coupled cells (146.7 ± 17 labeled cells per slice, n = 4) extending 349 ± 27 µm away from the recorded cell and homogeneously distributed in the outer border of the inner nuclear layer (average inter-somatic distance 12.9 ± 0.5 µm, Fig. 1c). We also found a group of cells that, despite sharing the morphological features of A17 cells (Fig. 1e), had a much higher R_in_ (2.4 ± 0.26 GΩ, n = 32, *p* < 10^−16^, Wilcoxon rank-sum test) and displayed no dye coupling. A histogram of R_in_ values observed in all recorded A17 cells shows two different cell populations that correlated with the presence (low R_in_) or absence (high R_in_) of dye coupling (Fig. 1f), suggesting that they may comprise two different classes of AC.

A distinctive characteristic of A17 cells is their expression of nicotinic cholinergic receptors, which can detect low acetylcholine (ACh) levels, induce inward currents and generate synaptic release^43^. Here, we confirm the cholinergic sensitivity of A17 cells and demonstrate that ACh can evoke inward currents in non-coupled A17s (ncA17), although they are about 3-fold smaller (average amplitude 196.8 ± 19.5 pA, n = 30 vs. 54.3 ± 5.4 pA, n = 26, for A17 and ncA17 respectively, *p* = 1.3 × 10^−8^, Wilcoxon rank-sum test, Fig. 1h), suggesting that neurochemical properties of these cell populations might also be similar. A17 cells showed strong and sustained inward currents after light onset (full-field light pulse, 3 s, 530 nm, average amplitude 84.5 ± 8.5 pA, n = 52, Fig. 1i), while ncA17 responded only weakly and in some cases also showed an inward current at the end of the light pulse (13 ON and 9 ON-OFF responses, average amplitude of ON phase 11.2 ± 1.6 pA, n = 22, *p* = 2.3 × 10^−11^ when compared to A17 cells, Wilcoxon rank-sum test Fig. 1h), indicating that either these cells differ in their presynaptic connectivity or that electrical coupling modulates their receptive field properties.

To understand the extent to which electrical coupling determines the physiological properties of A17 cells we evaluated the effect of pharmacological block of GJs. Currently, the identity of the connexin subunits forming GJs in A17 cells remains unknown, therefore we tested the effect of different connexin blockers on the R_in_ of A17 cells as a proxy for electrical coupling^44^. In A17 cells 18β-glycyrrhetinic acid (βGA) produced an ≈8-fold increase in R_in_ (from 0.2 ± 0.01 to 1.52 ± 0.17 GΩ, n = 12, *p* = 1.05 × 10^−5^, paired t-test, Fig. 1d, g). βGA perfusion required a substantial time to be effective (average perfusion time of 31 min) and its influence on R_in_ could be partially reversed after washout (from 1.12 ± 0.25 to 0.35 ± 0.08 GΩ, n = 6, average washout time 42 min). Mefloquine, a more specific GJ blocker^45–47^, had also a strong effect on the membrane resistance of A17 cells (Mef 50 µM, 29 min, from 0.25 ± 0.03 to 0.87 ± 0.2 GΩ, n = 6, *p* = 0.028, Wilcoxon signed-ranks test) while meclofenamic acid induced a significant but smaller increase in R_in_ (MFA 36 min, from 0.12 ± 0.02 to 0.6 ± 0.1 GΩ, n = 12, *p* = 0.0047, Wilcoxon signed-ranks test). On the other hand, carbenoxolone did not cause a significant change in R_in_ of A17 cells (Cbx 28 min, from 0.14 ± 0.02 to 0.18 ± 0.03 GΩ, n = 4, *p* = 0.14, Wilcoxon signed-ranks test, Fig. 1e). Perfusion of lanthanum and barium, Bupivacaine or tetrodotoxin (TTX) did not significantly change R_in_ of A17 cells (La^3+^ 100 µM + Ba^2+^ 1 mM, from 0.28 ± 0.03 to 0.28 ± 0.06 GΩ, n = 4, *p* = 0.46 Wilcoxon signed-ranks test; Bupivacaine 300 µM, from 0.15 ± 0.06 to 0.2 ± 0.05 GΩ, n = 4, *p* = 0.3, paired t-test; TTX 1 µm, from 0.27 ± 0.01 to 0.27 ± 0.01 GΩ, n = 12, p = 0.95, Wilcoxon signed-Ranks sum test), suggesting that connexin hemichannels, 2-pore leak K^+^ channels or Na^+^ channels respectively have little impact on the measured membrane conductance of this AC type. Similarly, blocking ATP dependent K^+^ channels and the CFTR anion transporter with Glibenclamide had no effect on A17 cell R_in_ (Glibenclamide 50 µM, from 0.16 ± 0.04 to 0.15 ± 0.04 GΩ, n = 5, *p* = 0.68, Wilcoxon signed-ranks test). These data therefore suggest that membrane resistivity of A17 cells is strongly influenced by the expression of connexin proteins. Blocking GJs also unmasked a voltage-dependent inward current (Fig. 1d, n = 12) whose voltage dependency and kinetics are similar to the L-type calcium currents previously described in A17 cells^48^. Interestingly, I-V curves of ncA17s (n = 18) resemble those from A17 cells in the presence of a GJ blocker, suggesting the expression of similar voltage-gated conductances in both cell groups (Fig. 1d). Blocking GJs in A17 cells also reduced the currents induced by activation of nicotinic ACh receptors to levels comparable to those observed in ncA17s in control conditions (βGA, from 171.4 ± 15 to 30.2 ± 6 pA, n = 12, *p* = 1.8 × 10^−6^, paired t-test; Mef, from 204.6 ± 45.3 to 10.3 ± 2.1 pA, n = 8, *p* = 0.017, Wilcoxon signed-ranks test, Fig. 1h). Light-induced currents in A17 cells were also impaired when electrical connections were blocked either using βGA (from 83.4 ± 14.7 to 17 ± 7.3 pA, n = 12, *p* = 0.0044, Wilcoxon signed-ranks test, Fig. 1i) or mefloquine (from 57.8 ± 16.9 to 26.5 ± 10.1 pA, n = 7, *p* = 0.028, Wilcoxon signed-ranks test), suggesting that light-evoked responses arise from the coordinated activation of a network of electrically coupled A17 cells. Finally, perfusion of βGA also reduced inward currents evoked by puff-applications of AMPA that activated glutamate receptors present in A17 cell varicosities^19^ (from 282 ± 89.6 to 163.4 ± 68.2 pA, n = 4, *p* = 0.033, Wilcoxon signed-ranks test, Fig. 1j). Therefore in rat retina, A17 cells exist in two varieties; Classical A17 cells, which have low R_in_, show dye-coupling to neighboring ACs^19,29^ and react strongly to excitatory stimuli, and ncA17 cells, which have high R_in_, show no dye-coupling and respond only weakly to direct pharmacological activation or light. These data parallels the existence of 2 classes of indoleamine accumulating cells in the rabbit (homologous  to rat and mouse A17 cells), which strongly differ in their degree of Lucifer Yellow diffusion through gap junctions^28^. Blocking the GJs of A17 cells changes their physiological properties to resemble those of ncA17 cells.

### Strong electrical coupling between A17 cells

To evaluate how electrical synapses can impact the function of A17 cells, we characterized their connectivity using paired whole-cell patch-clamp recordings. By inducing voltage steps in one of the patched cells (from -120 to 40 mV steps), electrical coupling, evidenced by a deflection in membrane current in the non-simulated cell, was detected every time an A17 cell pair was successfully targeted (54 pairs, average somatic distance 23.9 ± 2 µm, maximum distance 49.6 µm, Fig. 2b). In contrast, the induction of voltage steps in A17 cells had no influence when the paired cell was from a different AC class (7 recovered pairs, data not shown). In three cases an A17 and a ncA17 cell (average R_in_ 1.6 ± 0.2 GΩ, n = 3) were simultaneously recorded but no evidence of electrical coupling was observed, further indicating that they indeed comprise two distinct classes of AC, and showing that the two cell types can be found in the same slice under similar conditions. Using intracellular dyes of different colors we often observed colocalization in varicosities (Fig. 2a), therefore GJs might be located in close proximity to their chemical synapses (see also Li *et al*.^28^). Junctional conductance (G_j_) between pairs of A17 cells was on average 1.47 ± 0.2 nS (n = 54), covering a broad range from 0.01 to 5.9 nS (Fig. 2d). In current-clamp mode, positive current injection depolarized both A17 cells with an average coupling factor of 0.14 ± 0.08 (n = 8, Fig. 2c). Values of G_j_ were independent of which cell was stimulated (Fig. 2d), and the relationship between junctional current and voltage showed symmetrical rectification only when establishing a voltage difference greater than 80 mV, hence GJs between A17 might be formed by homologous connexin channels^49^ (Fig. 2e). Blocking GJs reduced the intercellular conductivity until it completely abolished electrical coupling (βGA decreased G_j_ from 0.74 ± 0.22 to 0.01 ± 0.01 nS, n = 8, *p* = 0.011, Wilcoxon signed-ranks test) and produced a correlated decrease in membrane conductance in both cells (Fig. 2f), further demonstrating the close relationship between intercellular coupling and membrane resistance in A17 cells. Indeed, there was a significant linear correlation between the average membrane conductance and the G_j_ from pairs of A17 cells (r = 0.75, p < 10^−6^, linear correlation test, Fig. 2g).

Finally, we characterized how electrical signals are transmitted between A17 cells. Measured G_j_ was unaffected by blocking Na^+^ channels (control G_j_ 0.64 ± 0.16 nS; TTX 1 µM 0.59 ± 0.14 nS, n = 5, *p* = 0.63, paired t-test), therefore suggesting that electrical signals do not require Na^+^ channels to propagate through GJs^26^. In addition, electrical transmission was neither facilitated nor depended on synaptic transmission, as blocking most ionotropic receptors of A17 cells had no significant influence in GJ coupling (G_j_ was 0.66 ± 0.16 and 0.73 ± 0.14 nS in control conditions and during block of synaptic receptors [see methods] respectively, n = 8, *p* = 0.27, paired t-test). The electrical filtering of signals when travelling from one cell to another via GJs was evaluated by injecting sinusoidal waveforms. Electrotonic conduction was strongly dependent on the oscillation frequency of the injected current, and at frequencies equal or higher than 1 Hz, there was a significant decrease in the coupling factor (e.g. CF was on average 0.1 ± 0.01 for steady state current and 0.029 ± 0.01 for a 25 Hz sinusoidal current injections, n = 6, Kruskal-Wallis and post-hoc Dunn’s test, Fig. 2h), and a concomitant increase in phase lag (e.g. signal lag was 88.8 ± 6.4 degrees for 25 Hz, n = 6). This strong attenuation, together with the absence of propagating sodium electrogenic activity to support signal transmission^26^, suggest that electrical synapses in A17 cells act mostly at a local level.

### Electrical coupling synchronizes A17 cells activity

**Figure 3:**
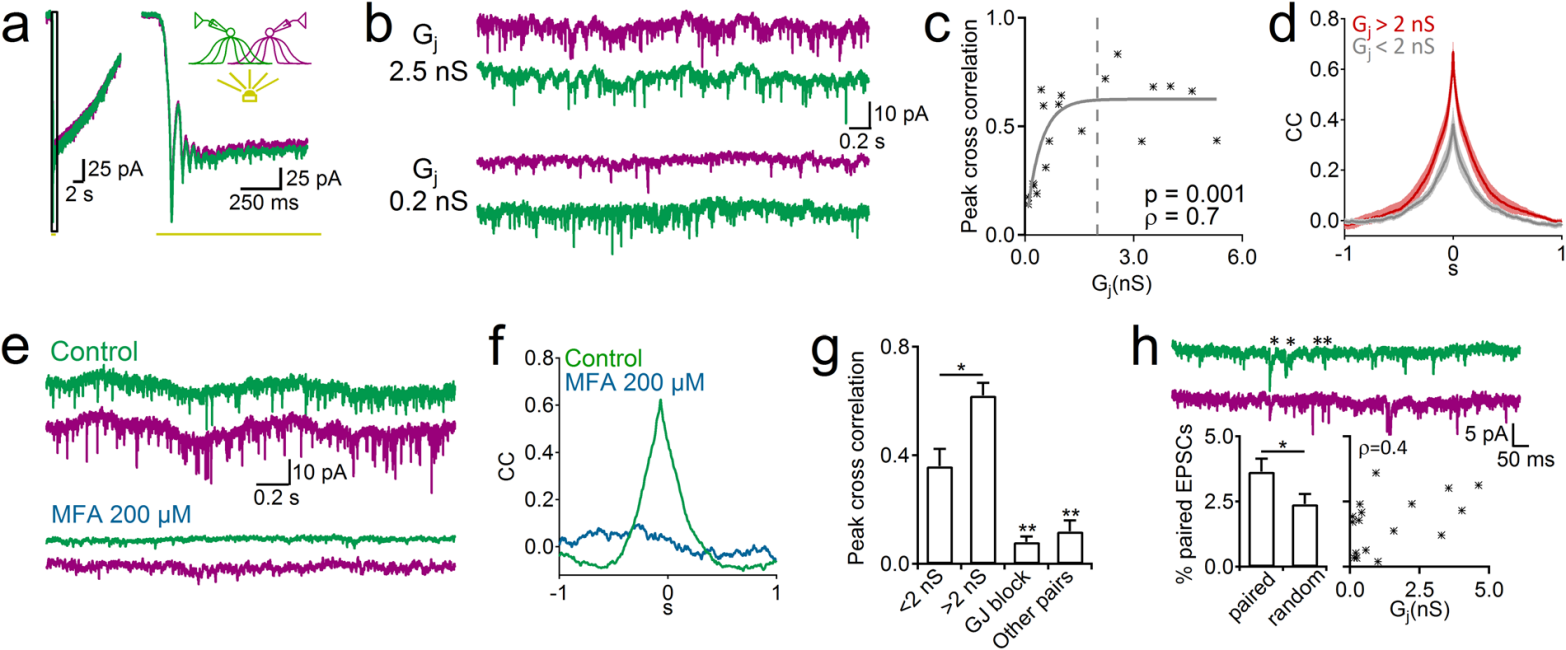
(**a**) Traces show the responses of two coupled A17 cells to a flash of light. Note the highly correlated signals of the magnified traces in the right part of the figure. (**b**) Recordings showing spontaneous activity in two electrically connected pairs of A17 cells with high (G_j_ = 2.5 nS, top traces) and low junctional conductance (G_j_ = 0.2 nS, bottom traces) (**c**) plot showing the relation between peak cross-correlation in spontaneous activity of A17 cells and the G_j_, dashed line represents the separation of two cluster of data (see text). (**d**) Average cross-correlogram of spontaneous activity signals from A17 pairs with high (>2 nS, n = 7, red trace) or low (<2 nS, n = 12, gray trace) junctional conductance. (**e**) Example recordings from a pair of coupled A17 cells (G_j_ = 4.6 nS) in control conditions and after perfusion with meclofenamic acid (MFA 200 µM). (**f**) Shows the cross-correlogram of recordings in figure e. (**g**) Summary plot of the cross-correlation of spontaneous activity for pairs with G_j_ < 2 (n = 12), G_j_ > 2 nS (n = 7), in pairs after blocking gap junctions (MFA 100–200 µM, n = 5) and from paired recordings between one A17 cell and another class of AC (n = 7). (**h**) Representative traces showing that only a percentage of EPSCs (asterisks) can be simultaneously observed (500 µs time window for co-occurring EPSCs) in a pair of coupled A17 cells. Bottom left, shows the percentage of events simultaneously occurring in pairs of A17 cells (pairs, n = 17) and in the same cells after shifting one of the cell recordings 2 ms (random). Bottom right, no significant monotonic correlation was found between G_j_ and the percentage of synchronized EPSCs.

What is the effect of such a highly interconnected network of A17 cells? As could be predicted for cells strongly coupled through GJs and sharing common inputs^6,25^, light responses in paired A17 cells were highly correlated (peak cross-correlation (CC) was 0.88 ± 0.04, n = 9, average G_j_ = 1.7 ± 0.5 nS, Fig. 3a) as well as spontaneous activity in the dark (CC 0.48 ± 0.05, n = 19, Fig. 3b–d,g) as previously described in mouse A17 cells^27^. The synchronization degree of spontaneous signals was significantly correlated to the strength of the electrical connection between A17 cell pairs (ρ = 0.7, *p* = 0.00097, Fig. 3b–c), and strongly connected pairs displayed a higher CC than those showing milder coupling (0.63 ± 0.05 vs. 0.39 ± 0.06 for pairs with G_j_ > 2nS and G_j_ < 2nS, cutoff selected using k-means clustering analysis, n = 12 and 7, *p* = 0.011, Wilcoxon signed-ranks test, Fig. 3d,g), suggesting that electrical synapses contribute to the generation of synchronous activity in the A17 cell network. Indeed, blocking electrical coupling by perfusing MFA (which reduced G_j_ from 2.1 ± 1 to 0.1 ± 0.1 nS, n = 5, *p* = 0.04, Wilcoxon signed-ranks test, data not shown) decreased the correlation of spontaneous synaptic activity (CC from 0.57 ± 0.1 to 0.08 ± 0.02, n = 5, *p* = 0.005, paired t-test, Fig. 3e–g). Pairs consisting of an A17 cell with another type of AC showed a significantly smaller cross-correlation (CC = 0.1 ± 0.04, n = 5, *p* = 0.0016, paired t-test, Fig. 3g) indicating that the high degree of synchronization occurs specifically between coupled A17 cells. On the other hand, although concurrent EPSCs were observed in some A17 cell pairs (Fig. 3h), analysis of spontaneous activity showed that only a small percentage of events arrived simultaneously (3.6 ± 0.5% of EPSCs occurred in both A17 cells with a lag of 0.5 ms), only slightly higher that the amount of randomly coincident events (2.4 ± 0.4% of EPSCs, n = 17, *p* = 0.016, paired t-test, Fig. 3f). The percentage of synchronized EPSPs was unrelated to electrical connectivity between cells (Spearman rank-order correlation coefficient ρ = 0.43, *p* = 0.08).

### GJ coupling between A17 cells facilitates reciprocal feed-back onto the RBCs

**Figure 4:**
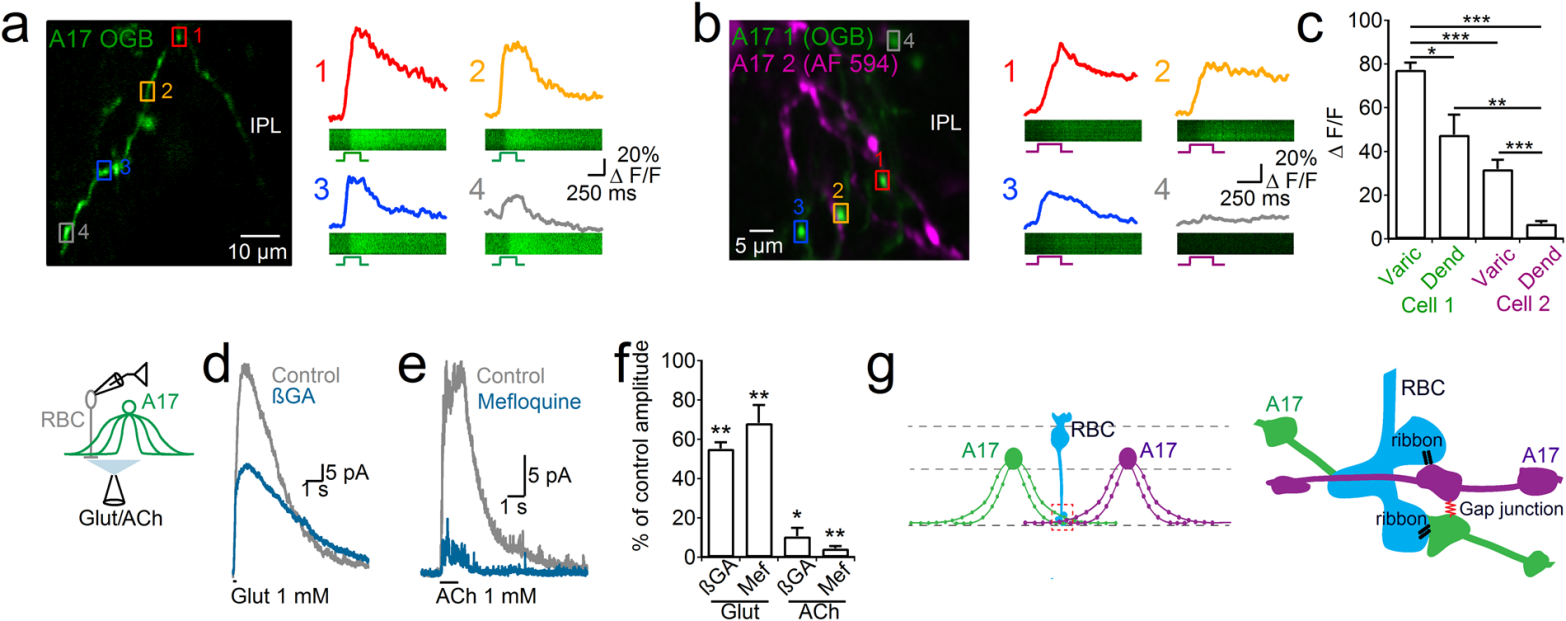
(**a**) 2-photon stack of one A17 cell dendrite filled with the indicator Oregon green BAPTA (OGB, 120 µM). On the right, Ca^2+^ transients evoked by a depolarization step (100 mV, 250 ms) at different regions of interest depicted by colored boxes on the left. (**b**) 2-photon projection of a representative sequential recording experiment. One A17 cell was filled with OGB and after loading the pipette was removed to maintain cell integrity. Afterwards a second A17 cell was patched with a solution containing Alexa fluor 594. Traces on the right depict Ca^2+^ transients in regions marked by boxes on the left image, evoked by depolarization of the second patched A17 cell (100 mV, 250 ms). (**c**) Summary plot shows the average change in fluorescence observed in an A17 cell varicosities or dendrites when the cell with OGB was stimulated either directly (green) or through the cell filled with Alexa Fluor 594 (magenta). (**d**) Left, scheme of the experimental setting in which glutamate or acetylcholine were puffed onto the inner plexiform layer to evoke GABA release from A17 cells, and the response was recorded in rod bipolar cells (RBCs). Glutamate was used in conditions in which feedback from A17 cells was isolated (TTX 1 µM, strychnine 2 µM). (**d**–**e**) Representative traces showing the inhibitory response observed in RBCs (holding voltage 0 mV) when either glutamate (d, 1 mM, 100 ms) or acetylcholine (e, 1 mM, 1 s) where puff-applied, in control conditions (gray traces) or after block of GJs (blue traces). (**f**) Summary plot showing the percentage of the response to glutamate or acetylcholine remaining after perfusion of GJ blockers (βGA 50 µM or mefloquine 50 µM). (**g**) Proposed functional structure of the electrically connected network of A17 cell reciprocal feedback synapses. ONL, outer nuclear layer; IPL, inner plexiform layer; INL, inner nuclear layer; GCL, ganglion cell layer.

How does electrical coupling influence the inhibitory function of A17 cells? During strong glutamatergic depolarization^22,48^ or after cholinergic activation of A17 cell synapses^43^, voltage-dependent Ca^2+^ channels (VGCCs) induce GABA release. Therefore, depolarization of one A17 cell might facilitate GABA release in coupled neighboring cells by promoting VGCC activation. To support this hypothesis, we tested if the depolarization of one A17 cell can induce an intracellular Ca^2+^ increase in closely located neighbors. Patch-clamp recordings were established using the Ca^2+^ indicator Oregon green BAPTA (OGB) in one A17 cell. Voltage steps (100 mV, 250 ms) in the cell loaded with OGB induced robust Ca^2+^ transients whose amplitude was larger in varicosities than in dendrites (77.4 ± 6.8 vs. 47.7 ± 9.1 ΔF/F, 28 varicosities and 9 dendritic locations, n = 4 cells, *p* = 0.04, Wilcoxon rank-sum test, Fig. 4a,c), supporting the presence of VGCCs in A17 cell varicosities^26,48^. After the loading period, the pipette was slowly removed until the membrane re-sealed in order to maintain the integrity of the cell. Afterwards, another A17 cell was patched using a fluorescent dye that did not permeate through GJs (Alexa Fluor 594). Remarkably, when this second cell was depolarized, Ca^2+^ transients were observed in varicosities of the cell containing OGB (Fig. 4b–c). Ca^2+^ signals were smaller when evoked by stimulation of the second cell (32 ± 3.6 ΔF/F, 38 varicosities, 5 cells, *p* = 1.66 × 10^−7^, Wilcoxon rank-sum test), and were specifically located in varicosities (6.8 ± 1.1 ΔF/F, 14 dendritic locations, 3 cells, *p* = 1.54 × 10^−5^, Wilcoxon rank-sum test), showing that depolarization of one A17 cell is capable of inducing Ca^2+^ elevations in varicosities of adjacent A17 cells.

Finally, to test whether GJ coupling can facilitate GABAergic control of RBC depolarization, we studied the effect of connexin blockers on agonist-induced neurotransmitter release from A17 onto RBCs. Puffs of glutamate were applied under conditions in which the contribution of A17 cells to GABAergic feedback can be isolated from inhibition provided by other ACs (TTX 1 µM, strychnine 2 µM)^19,43^. Perfusion with βGA diminished glutamate-evoked IPSCs to almost half of the original response (βGA 50 µM, 55.5% of control, n = 7, *p* = 0.0014, paired t-test, Fig. 4d,f). This reduction was significantly larger (*p* = 0.006, unpaired t-test) than the effect of βGA on GABA-induced currents in RBCs (80 ± 3% of control after βGA perfusion, n = 6, *p* = 0.0035 paired t-test). Perfusion of mefloquine produced a similar reduction in glutamate-induced GABA release from A17 cells (Mef 50 µM, 68.4 ± 5.3% of control, n = 5, *p* = 0.0088, paired t-test, Fig. 4) without having a direct effect on RBC GABA receptors (100.8 ± 2% of control, n = 6, *p* = 0.67, paired t-test). Therefore, GJ blockers reduced glutamate-induced GABA release from A17 cells probably by decreasing coupling between A17 cells. When ACh was used to predominantly stimulate VGCC-mediated GABA release from A17 cells^43^, blocking GJs with βGA or Mef produced a strong reduction in the evoked response (reduction to 10.6 ± 3.1 and 4.4 ± 1.3% of control for βGA and Mef 50 µM, n = 4 and 7, *p* = 0.04 and 0.009 respectively, Wilcoxon rank-sum test, Fig. 4e,f). These results suggest that coupling between A17 cells supports reciprocal feedback to a single RBC.

## Discussion

A17 cells are a fundamental part of the network for scotopic vision^20,21,24^ and the second most numerous AC in the mammalian retina^50^. The scotopic circuit exhibits a high gain which allows the transformation of single photon detections into RGC action potentials^1^. Synchronization might be therefore critical to keep low noise levels and ensure reliable signal propagation. Here, we provide a detailed description of electrical coupling between A17 cells and the role it plays in signal processing within the scotopic pathway.

### Most A17 cells are electrically interconnected

Electrical synapses are widespread in the retina, expressed in different cell types and enhance the signal processing capabilities of the network^34–38^. In the rat retina, we have found two classes of A17 cells that differ in their electrical coupling degree similar to their homologous ACs in the rabbit retina^28,42^, suggesting a functional role for the presence of strongly and weakly coupled A17 cells. Studying their specific synaptic connectivity could help to understand their differential contribution to retinal information processing. In our dark-adapted conditions, coupled A17 cells presented a low membrane resistance^19,43^, lower than non-A17 ACs unintendedly recorded in this and other studies (2.2 ± 0.2 GΩ, n = 138 ACs)^43^. Since blocking GJs increases R_in_ of A17s more than 8-fold and considering that ncA17 cells displayed a 10 times larger R_in_, we propose that measured conductivity of A17 cells is largely determined by their intercellular coupling, similar to observations in AII ACs^44^. Although the narrow A17 cell dendrites are likely to induce an underestimation in the measured coupling^23,26^, A17 GJs  had a larger junctional conductance than most dendro-dendritic electrical synapses between GABAergic interneurons^30,51,52^, can conduct large molecules like Lucifer Yellow^28,29^, and directly connect A17 cells to approximately 51 partners in the rabbit^28^, showing that these cells form a tightly interconnected electrically coupled network. Interestingly, blocking GJs reduced the responses to light or direct pharmacological activation of ionotropic receptors in A17 cells, despite a concomitant increase in R_in_ which should generate larger voltage changes after stimulation. This observation suggests that synaptically evoked signals propagate between A17 cells and increase the net response recorded in a single cell. Indeed, our imaging experiments indicate that Ca^2+^ signals canbe evoked by neighbouring  A17 cells, a mechanism by which GJs might enhance signal synchronization.

The identity of connexins proteins expressed in A17 cells is unknown. Cx36 immunolabeling can be found in the inner portion of the IPL^36,53,54^ but does not colocalize with A17 dendrites^36^ and carbenoxolone, an effective Cx36 blocker^55^, had no effect on R_in_ of A17 cells. Therefore, another connexin protein expressed in the IPL, such as Cx45^53,56,57^, may form the building blocks for A17 cell electrical synapses. Experiments using knockout mice for different connexins are required to determine the precise subtypes mediating coupling between A17 cells.

### Neighboring varicosities from different A17 cells are synchronized through GJs

Previous reports have suggested that synchrony between A17 cells arises from sharing the same excitatory inputs^27^. Although we also observed that light-evoked or spontaneous signals are highly correlated between A17 cells, several observations suggest that electrical coupling plays also an important role in synchronizing voltage fluctuations between varicosities from different A17 cells: First, we observed a correlation between coupling strength and the degree of cross-synaptic synchrony (Fig. 3c). That this correlation is not linear may be due to contributing factors like the presence of shared inputs^27^. Second, blocking GJs eliminated synchrony (Fig. 3e–f). Although reduction of excitatory inputs alone disrupts A17 cell synchrony^27^, this manipulation can also conceal the suggested contribution of GJs to synchrony by decreasing the amplitude of synaptically generated voltage changes. Third, we did not observe a significant amount of coincident EPSCs between pairs of A17 cells (Fig. 3h) as it should be expected if cross-synchrony depended only on the existence of shared inputs with high release probability^27^. In contrast, filtering through electrical synapses could explain the correlated spontaneous activity without synchronous EPSCs. Discrepancies between our observations and others^22,27^ could be due to animal species differences (rat vs. mouse), age (>P30 vs. P17-21) or even slice preparation methodology. In light-adapted rat or mouse retina, dye coupling between A17 cells has not been reported^19,26,48^, suggesting that it is affected by the illumination conditions^34,58^. Interestingly, we observed a 10 times larger average G_j_ than previously reported^27^ which could explain the greater role of GJs in cross-synchronization between A17 cells in our preparation

Ca^2+^ imaging experiments have also shown that unitary synaptic signals generated in a varicosity are rarely capable to activate VGCCs in adjacent varicosities of the same A17^26^. Here, we observed that direct somatic depolarization of A17 cells (>80 mV) induces a rise in intracellular Ca^2+^ in the stimulated cell as well as in neighboring A17 cells. Our data and prior studies^28^ suggest that electrical synapses are located in the vicinity of synaptic contacts, a position ideally suited to convey signals between varicosities with minimum attenuation. Because the junctional conductance is large compared to the “true” membrane resistivity (after blocking GJs), electrical synapses rather than the thin A17 dendrites might provide the main route for charge flow after light activation. This could favor electrical isolation of varicosities from the same cell^26^, while simultaneously distributing the signal over the ensemble of neighboring varicosities from coupled A17 cells.

Although spontaneous voltage fluctuations of RBCs need to be minimal in the dark in order to achieve single photon detection, this is no longer a requirement in dim-light, and indeed correlation between A17 cells decreases as luminosity increases^27^. Modulation of electrical coupling could provide a mechanism to dynamically regulate signaling synchrony between A17 cells. Electrical synapses are widely known to increase synchrony between electrically coupled GABAergic cells^31,33,35,51,59–61^, and in A17 cells they are especially fit to do so given their subcellular location and biophysical properties.

### Coupling of neighboring varicosities enhances feedback onto RBCs

Due to the high convergence and supra-linearity of the scotopic pathway, spontaneous synaptic fluctuations can heavily influence signal-to-noise ratio when photons are sparse^1^. Signals from one rod are transmitted to ≈10 different AII cells as the circuits diverges^62^. Glutamate release from RBC to AII cells is fast and transient^63^ due to a high release probability and small ready-releasable pool^64,65^. A17 reciprocal feedback curtails RBC light responses^20,21^, accelerating rod signals and regulating short-term depression at the RBC-AII synapse^22^. In order to maintain a synchronous retinal output, it would be advantageous for inhibitory reciprocal synapses onto a given RBC to behave synchronously; a difficult task since each of them arises from a different A17 cell. Electrical coupling between varicosities surrounding one RBC axon could therefore homogenize voltage and Ca^2+^ fluctuations generated at individual varicosities, thus controlling in a similar manner synaptic release and ready-releasable pool size for different RBC-AII synapses (Fig. 4g). Such an organization is supported by the effect of GJ blockers onto GABA release from A17 cells. Upon glutamate activation, electrical synapses between A17 varicosities could facilitate GABA release from coupled terminals by enabling either electrotonic spread of depolarization and subsequent VGCCs recruitment or by Ca^2+^ diffusion. In this way, GJ coupling could coordinate signal integration at varicosities contacting closely located RBCs, enhancing efficiency and reducing noise from the feedback provided to the RBC population.

Similar to A17 cells, HCs provide feedback inhibition to cone photoreceptors and are interconnected through GJs that allow lateral signal propagation^66^. Remarkably, A17 cells have been proposed to generate the center-surround organization of AII cell receptive fields^67^ just as HCs do at the bipolar cell level. Although the lack of regenerative events and the thin diameter of its dendrites suggest a local function for A17 reciprocal feedback^26^, we have shown that significant amount of current can flow laterally through GJs. Interestingly, HCs might fulfil both, a spatially restricted as well as an extended inhibitory function in retinal processing^66^. It would be interesting to investigate if A17 cells also have a dual role in controlling RBC-AII activation.

In summary, we have shown that A17 amacrine cells of the rat retina are strongly interconnected by electrical synapses forming a network that synchronizes activity and enhances reciprocal feedback onto RBCs, thereby shaping visual flow through the classic scotopic pathway.

## Material and Methods

Retinal slices were obtained from adult Sprague Dawley rats (30–50 days old) of both sexes. After a >1 h of dark adaptation and under dim-red light, animals were deeply anesthetized and decapitated, eyes were removed and submerged in artificial cerebrospinal fluid (ACSF) containing (in mM), 119 NaCl, 23 NaHCO3, 1,25 NaH2PO4, 2,5 KCl, 2,5 CaCl2, 1,5 MgCl2, 20 Glucose, 2 Na-Pyruvate, 1 ascorbic acid (equilibrated with 95% 0_2_ and 5% C0_2_). The retina was embedded in agarose and 200 µm sections were obtained using a vibratome. Slices were kept in a dark chamber at room temperature where they remained light-responsive for about 8 hours after dissection. Experimental procedures were approved by the bioethics committee of the Universidad de Valparaíso and in accordance with the bioethics regulation of the Chilean Research Council (CONICYT). Cells were selected under infrared DIC microscopy. Patch pipettes were pulled from borosilicate glass to resistances of 5 MΩ or 10 MΩ (for AC and BC recordings, respectively) when filled with an intracellular solution containing (mM) 125 cesium methanesulfonate, 10 HEPES, 5 EGTA, 6 Na_2_ATP, 0.4 GTP, 15 TEA-Cl, 1 MgSO_4_ and 1% Lucifer Yellow or 100 µM Alexa 488, Alexa 568 Alexa 594 or neurobiotin. In a subset of experiments pipettes were front-filled with dye-free solution, and mild positive pressure was always used when approaching the target cell (≈10 mbar) to avoid unspecific labelling of cells through dye spillover. Electrical signals were recorded using either a PC-501A (Warner Instruments), EPC 7 plus or an EPC 10 amplifier (HEKA Elektronik), digitized at 20 kHz (PCI-6221, National Instruments or LIH 8 + 8, HEKA) and stored using custom software written in IGOR PRO (Wavemetrics) or Patch Master (HEKA). Series resistance was always below 25 or 30 MΩ for ACs and RBs respectively, and left uncompensated. Liquid junction potential was ≈10 mV and corrected off-line. ACs were held at a membrane potential of −60 mV and IPSCs in RBCs were recorded at 0 mV. For blocking synaptic transmission slices were perfused with ACSF supplemented with a combination of ionotropic receptor antagonists (NBQX 10 µM, TPMPA 50 µM, Gabazine 5 µM and Strychnine 2 µM). For Ca^2+^ imaging, an intracellular solution containing (in mM) 115 K-gluconate, 20 KCl, 10 HEPES, 2 MgCl_2_, 10 Na_2_-phosphocreatinine and 0.12 Oregon Green Bapta (pH 7.2) was used. Ca^2+^ signals were acquired at 25–100 Hz using an epifluorescence (Zeiss Axioexaminer with RedShirt neuroCCD camera) or a 2 photon system (Femto Alba, Femtonics). Data of Fig. 4 were obtained from two sequential recordings using epifluorescence and 5 two-photon microscopy recordings. Drugs were prepared as stock solutions in bi-distilled water or DMSO and were diluted to the final concentration before the experiment. For localized applications, drugs were dissolved in HEPES-buffered ACSF and applied from glass pipettes using a Pico-spritzer. Light stimulation was achieved using a LED light source of 530 nm wavelength focused through the microscope objective. Measured light intensity of the flash stimulus was 1.9 log scot cd s m^−2^ with a wavelength of 500 nm. Data analysis was performed in IGOR PRO. R_in_ was obtained by fitting a line to the current responses to voltage steps (10 mV) from −120 to −80 mV. For obtaining the gap junctional conductance (G_j_) and current (I_j_), voltage steps (−120 to + 60 mV) were applied separately in both cells, and values of G_j_ and I_j_ were calculated at every step by considering voltage drops caused by series resistance of both recording electrodes and current leak through the cell membrane^68^. The frequency dependence of electrical transmission between A17 cells was measured by injecting sinusoidal current and calculating the power ratio at the stimulation frequency. Phase lag was estimated as the deviation from 0 of the voltage response cross-correlogram, divided by the period of the stimulus waveform and multiplied by 2π. Cross-correlation of spontaneous activity was evaluated from 60–180 s of data which was low-pass (100 Hz) and band-reject (49–51 Hz) filtered. Cross-correlation was calculated from 7.5 s bins and averaged. Resulting cross-correlograms were divided by the standard deviation of signals obtained in both cells and by the total number of points. Clustering in Fig. 3c was performed using the k-means algorithm defining a maximum of two clusters. For studying the co-occurrence of EPSCs between pairs of A17 cells, spontaneous events were detected using the Neuromatic IGOR PRO package (www.neuromatic.thinkrandom.com) in both cells and the proportion of EPSCs found in both cells inside a 0.5 ms time window was calculated. Results were tested against the correlated EPSCs found in the same traces after offsetting the recordings of one cell by 2 ms. Data are shown as average ± standard error. Statistical analysis was performed using Microsoft excel plugin real statistics (www.real-statistics.com). If Shapiro-Wilk and F-test indicated normal and covarying distributions, respectively, statistical significance was evaluated using two-tailed student’s t-test. Otherwise a Wilcoxon rank-sum test or a Wilcoxon signed-ranks test for pairwise comparisons was used. Multiple-group data was first analyzed with a Kruskal-Wallis test followed by a Dunn’s test for pairwise comparisons. Spearman’s rank correlation coefficient (ρ) was used to detect the degree of monotonic correlation. *Indicates p < 0.05, **p < 0.01, and ***p < 0.001.
