# Publisher Correction: Electrical coupling between A17 cells enhances reciprocal inhibitory feedback to rod bipolar cells

**DOI:** 10.1038/s41598-018-24193-6

**Published:** 2018-04-17

**Authors:** Claudio Elgueta, Felix Leroy, Alex H. Vielma, Oliver Schmachtenberg, Adrian G. Palacios

**Affiliations:** 1 Centro Interdisciplinario de Neurociencia de Valparaíso, Universidad de Valparaíso, Valparaíso, Chile; 2 Physiology Institute I, Alberts Ludwig University, Freiburg, Germany; 3 Neuroscience department, Columbia University Medical Center, 1051 Riverside Drive, New York, NY 10032 USA

Correction to: *Scientific Reports* 10.1038/s41598-018-21119-0, published online 15 February 2018

In the original version of this Article, Alex H. Vielma, Oliver Schmachtenberg and Adrian G. Palacios were incorrectly affiliated with ‘Physiology Institute I, Alberts Ludwig University, Freiburg, Germany’. The correct affiliation is listed below.

Centro Interdisciplinario de Neurociencia de Valparaíso, Universidad de Valparaíso, Valparaíso, Chile.

Additionally, the Acknowledgements section in this Article was incomplete.

“This study was supported by CINV, a Millennium Institute (P09-022-F) funded by the Millenium Scientific Initiative of the Ministry of Economy, Development and Tourism (Chile), a graduate MECESUP fellowship and a CONYCIT PhD support grant to CE and a FONDECYT No. 1171228. We thank Dr. Pepe Alcami, Dr Michael Strüber and Dr. Shakuntala Savanthrapadian for their comments and suggestions on the manuscript.”

now reads:

“This study was supported by CINV, a Millennium Institute (P09-022-F) funded by the Millenium Scientific Initiative of the Ministry of Economy, Development and Tourism (Chile), a graduate MECESUP fellowship and a CONYCIT PhD support grant to CE and a FONDECYT No. 1171228 and 1150638. We thank Dr. Pepe Alcami, Dr Michael Strüber and Dr. Shakuntala Savanthrapadian for their comments and suggestions on the manuscript.”

